# Patients' and Caregivers' Suggestions for Improving Assisted Dying Regulation: A Qualitative Study in Australia and Canada

**DOI:** 10.1111/hex.14107

**Published:** 2024-06-19

**Authors:** Ruthie Jeanneret, Eliana Close, Lindy Willmott, Jocelyn Downie, Ben P. White

**Affiliations:** ^1^ Australian Centre for Health Law Research, Faculty of Business and Law Queensland University of Technology Brisbane Queensland Australia; ^2^ Faculties of Law and Medicine, Health Law Institute Dalhousie University Halifax Nova Scotia Canada

**Keywords:** assisted dying, caregivers, medical assistance in dying, patient experience, regulation, suggestions to improve systems, voluntary assisted dying

## Abstract

**Introduction:**

Assisted dying (AD) has been legalised in a small but growing number of jurisdictions globally, including Canada and Australia. Early research in both countries demonstrates that, in response to access barriers, patients and caregivers take action to influence their individual experience of AD, as well as AD systems more widely. This study analyses how patients and caregivers suggest other decision‐makers in AD systems should address identified issues.

**Methods:**

We conducted semistructured, qualitative interviews with patients and caregivers seeking AD in Victoria (Australia) and three Canadian provinces (British Columbia, Ontario and Nova Scotia). Data were analysed using reflexive thematic analysis and codebook template analysis.

**Results:**

Sixty interviews were conducted with 67 participants (65 caregivers, 2 patients). In Victoria, this involved 28 interviews with 33 participants (32 caregivers, 1 patient) about 28 patient experiences. In Canada, this involved 32 interviews with 34 participants (33 caregivers, 1 patient) about 33 patient experiences. We generated six themes, corresponding to six overarching suggestions by patients and caregivers to address identified system issues: (1) improved content and dissemination of information about AD; (2) proactively develop policies and procedures about AD provision; (3) address institutional objection via top‐down action; (4) proactively develop grief resources and peer support mechanisms; (5) amend laws to address legal barriers; and (6) engage with and act on patient and caregiver feedback about experiences.

**Conclusion:**

AD systems should monitor and respond to suggestions from patients and caregivers with firsthand experience of AD systems, who are uniquely placed to identify issues and suggestions for improvement. To date, Canada has responded comparatively well to address identified issues, whereas the Victorian government has signalled there are no plans to amend laws to address identified access barriers. This may result in patients and caregivers continuing to take on the burdens of acting to address identified issues.

**Patient or Public Contribution:**

Patients and caregivers are central to this research. We interviewed patients and caregivers about their experiences of AD, and the article focuses on their suggestions for addressing identified barriers within AD systems. Patient interest groups in Australia and Canada also supported our recruitment process.

## 1 Introduction

Assisted dying (AD) has been legalised in a small but growing number of jurisdictions globally, including Canada and Australia [1]. In Canada, a federal legislative framework for AD was introduced via Bill C‐14 in 2016 [2, 3], and amended in 2021 by Bill C‐7 [4]. All six Australian states have passed laws since 2017 [5]. Australia also has two territories: the Northern Territory is currently considering AD law reform, and the Australian Capital Territory passed a law to permit AD on 5 June 2024 [6, 7]. AD is referred to as ‘medical assistance in dying’ (MAiD) in Canada, and ‘voluntary assisted dying’ (VAD) in Australia. We use the term AD, referring to when a person seeks assistance from a health practitioner to receive medication that will end their life.

The importance of involving patients and caregivers in decision‐making about their own healthcare is well‐established [8]. Patients' and caregivers' potential to improve healthcare *systems*, including involvement in designing and evaluating those systems beyond their own experience, is also increasingly recognised [8, 9]. Patients and caregivers may make ‘tenacious, unofficial efforts’ to address safety issues [10], make complaints [11], report adverse events to official bodies [9, 12, 13], undertake litigation [14] or a variety of other actions [9, 10]. This phenomenon of patients and caregivers influencing healthcare design and delivery has been described variously as patient/consumer/caregiver involvement, engagement or participation [15, 16]. Some scholars argue that these efforts constitute ‘regulation’ (i.e., shaping or steering the system) [17, 18, 19].

In the AD context, patients and caregivers have influenced their own experiences of AD through actions such as selectively choosing a provider or health service or advocating about their care [18]. They also take actions that influence wider AD *systems* in ways that have been characterised as ‘regulatory’, including storytelling, making complaints, providing feedback and law reform efforts [18, 19]. These actions to influence AD systems relate to identified system issues within Australian and Canadian systems, including overly restrictive legal requirements [20, 21, 22, 23, 24], institutional objections [25, 26, 27] and a lack of awareness and information about AD and care pathways [20, 21, 22, 28, 29, 30].

While some patients and caregivers willingly engage in actions to improve AD systems and derive meaning from their efforts [19], healthcare systems should ensure the burden of taking action to address system issues does not fall on patients and caregivers [10, 17, 31]. Patients and caregivers may be unwilling [32, 33], or unable, to act in this way. Opportunities for patients and caregivers to share stories, feedback and suggestions should be provided. However, AD systems should not expect patients and caregivers to go beyond this, particularly given how unwell patients seeking AD are and the grief that caregivers may be experiencing during and after AD. Other key decision‐makers in AD systems, such as policymakers, government officials, healthcare institutions and healthcare professionals should monitor and respond appropriately to patients' and caregivers' suggestions to improve AD systems.

Because of their expertise as users of AD systems, patients and caregivers are uniquely placed to (1) identify issues in AD systems and (2) make suggestions for how system issues can be addressed. The aims of this article are therefore twofold. The first aim is to outline system issues in Canada and Australia that patients and caregivers are seeking to address through their own actions. The second aim is to articulate patients' and caregivers' suggestions for how *other* actors within AD systems could respond to better address these system issues. This research seeks to extend knowledge by providing concrete suggestions from patients and caregivers about how AD system issues can be addressed. By synthesising data from Canada and Australia to identify suggestions that resonate across both jurisdictions, this research aims to provide insights into AD systems in other countries and may be particularly useful for jurisdictions reviewing AD systems or those considering legalising or implementing AD.

## 2 Australian and Canadian Context

The focus of our study is on the Australian state of Victoria and the federal Canadian framework (Quebec has a distinct legislative history) [3]. Australia and Canada have been chosen for this analysis because, of jurisdictions with AD systems, they are similar in terms of culture, political, legal and health system structures, geographical size and population distribution. Canada has also been recognised as a particularly useful comparator in Australia when debating and implementing AD systems locally [34]. Victoria was chosen because it was the first Australian state to legalise AD and all states generally follow the conservative Victorian model, with some variation [5].

Victorian and Canadian AD systems share some similarities: both currently limit access to adults suffering intolerably who are assessed as eligible by at least two independent practitioners [2, 4, 35]. But there are many differences. We provide an overview of key aspects of Victoria's and Canada's laws in Table 1 and highlight three aspects of the wider ‘regulatory’ landscape (i.e., factors influencing the operation of AD beyond the law) below.

**Table 1:** Key aspects of AD laws in Canada and Australia.

	Canada Bill C‐14 [2] (from 17 June 2016, amended by Bill C‐7 in March 2021)	Canada Bill C‐14, as amended by Bill C‐7: Track 1 (natural death reasonably foreseeable) [4] (from 17 March 2021)	Canada Bill C‐14, as amended by Bill C‐7: Track 2 (natural death not reasonably foreseeable) [4] (from 17 March 2021)	Victoria Voluntary Assisted Dying Act 2017 [35] (from 19 June 2019)
1 Eligibility requirements				
1.1 Age	Aged 18 or over	Aged 18 or over	Aged 18 or over	Aged 18 or over
1.2 Decision‐making capacity	Capable of making decisions with respect to their health	Capable of making decisions with respect to their health	Capable of making decisions with respect to their health	Have decision‐making capacity in relation to voluntary assisted dying
1.3 Residency requirements	Eligible for health services funded by a Canadian government (or eligible, but for a waiting period)	Eligible for health services funded by a Canadian government (or eligible, but for a waiting period)	Eligible for health services funded by a Canadian government (or eligible, but for a waiting period)	Australian citizen or permanent resident, and ordinarily resident in Victoria for 12 months at time of making their first request for AD
1.4 Eligible medical condition	Have a grievous and irremediable medical condition, i.e.: − a serious and incurable illness, disease or disability − be in an advanced state of irreversible decline in capability − the illness or state of decline is causing enduring intolerable physical or psychological suffering that cannot be relieved under conditions deemed acceptable by the person − natural death has become reasonably foreseeable	Have a grievous and irremediable medical condition, i.e.: − a serious and incurable illness, disease or disability (excluding mental illness) − be in an advanced state of irreversible decline in capability − the illness or state of decline causes enduring intolerable physical or psychological suffering that cannot be relieved under conditions deemed acceptable by the person	Have a grievous and irremediable medical condition, i.e.: − a serious and incurable illness, disease or disability (excluding mental illness) − be in an advanced state of irreversible decline in capability − the illness or state of decline causes enduring intolerable physical or psychological suffering that cannot be relieved under conditions deemed acceptable by the person	Diagnosed with a disease, illness or medical condition that is: − incurable − advanced, progressive and will cause death − is expected to cause death within 6 months (or 12 months for neurodegenerative conditions) − is causing suffering to a person that cannot be relieved in a manner the person considers tolerable
1.5 Timeframe to death	Natural death has become reasonably foreseeable	Whether natural death has become reasonably foreseeable determines whether someone is on Track 1 or Track 2, but not an eligibility criterion	Whether natural death has become reasonably foreseeable determines whether someone is on Track 1 or Track 2, but not an eligibility criterion	6 months (or 12 months for neurodegenerative conditions)
1.6 Mental illness/disorder as the sole underlying medical condition	Unable to access AD	Currently unable to access AD	Currently unable to access AD	Unable to access AD
1.7 Consent	Provides informed consent to receive AD after having been informed of the means that are available to relieve their suffering, including palliative care	Provides informed consent to receive AD after having been informed of the means that are available to relieve their suffering, including palliative care	Provides informed consent to receive AD after having been informed of the means that are available to relieve their suffering, including palliative care They must also be informed, by the first and second assessors/providers, of means available to relieve suffering, including counselling services, mental health and disability support services, community services and palliative care and offered consultations with relevant professionals who provide those services or care There must also be agreement between the first and second assessor/provider that the person has given serious consideration to means available to relieve suffering (See also 2.12 information requirements below)	Not an eligibility criterion but consent is required as part of request and assessment process (the person must make multiple requests for AD) The person must also understand specific information, including about palliative care
1.8 Voluntariness	Make a voluntary request for AD, free from external pressure or influence	Make a voluntary request for AD, free from external pressure or influence	Make a voluntary request for AD, free from external pressure or influence	Not an eligibility criterion, but whether a person's request is voluntary and free from coercion is assessed during the request and assessment process
2 Request/assessment process and safeguards				
2.1 Initiating discussions about AD	No prohibition on who can initiate discussions about AD	No prohibition on who can initiate discussions about AD	No prohibition on who can initiate discussions about AD	Registered health practitioners prohibited from initiating discussions about AD in the course of providing health care or professional care services
2.2 Requests for AD	One written request	One written request	One written request	A first request (verbal, written or other means), a written declaration (in writing) and a final request (verbal, written or other means)
2.3 Witnessing requirements	Written request must be witnessed by two independent, eligible witnesses	Written request must be witnessed by one independent, eligible witness	Written request must be witnessed by one independent, eligible witness	Written declaration must be witnessed by two independent, eligible witnesses
2.4 Enduring request	Person must be informed by both assessors that they may, at any time and in any manner, withdraw their request	Person must be informed by both assessors that they may, at any time and in any manner, withdraw their request	Person must be informed by both assessors that they may, at any time and in any manner, withdraw their request	Person's request must be enduring, which is assessed by each of the two assessing practitioners during the first and consulting assessments, at the time of applying for a permit for administration, and at the time of administration
2.5 Eligibility assessments	Two independent medical practitioners and/or nurse practitioners must independently assess the person and confirm they meet all eligibility criteria	Two independent medical practitioners and/or nurse practitioners must independently assess the person and confirm they meet all eligibility criteria	Two independent medical practitioners and/or nurse practitioners must independently assess the person and confirm they meet all eligibility criteria	Two independent medical practitioners must independently assess the person and confirm they meet all eligibility criteria and understand specific information
2.6 Specialist requirement	No specialist requirement	No specialist requirement	Consultation requirement: if neither of the assessing medical practitioners and/or nurse practitioners has expertise in the condition causing the person's suffering, they must consult with a medical practitioner or nurse practitioner who has that expertise	One medical practitioner must have expertise and experience in the person's disease, illness or medical condition (interpreted as requiring a specialist)
2.7 Use of telehealth allowed	Yes	Yes	Yes	No (due to policy enacted by the Victorian Government [36], in response to the *Criminal Code Act 1995* [Cth]) [37]
2.8 Waiting period	10 days between written request and provision (can be shortened if death or loss of capacity to consent imminent)	No waiting period	90 days between first assessment and provision (can be shortened if loss of capacity to consent is imminent)	9 days between the first request and final request (and there must be at least one day between the consulting assessment and final request) (can be shortened if death is imminent)
2.9 Final consent immediately before administration	Final consent required to be provided (i.e., person must have decision‐making capacity at the time they provide final consent)	Final consent required to be provided unless an exception applies	Final consent required to be provided unless an exception applies	For practitioner administration, the person may make a request for administration of the medication, and the medication may be administered, only if the person has decision‐making capacity at the time of the request and administration For self‐administration, the person must have decision‐making capacity to request that a self‐administration permit be applied for
2.10 Exception to final consent requirement	No exception	Provider‐administered MAiD: Final consent not needed if the criteria are met for a waiver of final consent (a written arrangement entered into before loss of capacity). The substance can be administered on the terms of the arrangement, unless the person demonstrates, by words/sounds/gestures refusal to have the substance administered or resists administration Self‐administered MAiD: Final consent not needed if advance consent for provider‐administered MAiD after failed self‐administration (i.e., a written agreement entered into before loss of capacity) exists	Provider‐administered MAiD: Final consent must be provided (a waiver of final consent is unavailable) Self‐administered MAiD: Final consent not needed if advance consent for provider‐administered MAiD after failed self‐administration (i.e., a written arrangement entered into before loss of capacity) exists	No exception
2.11 Method of administration	Provider‐administered or self‐administered allowed	Provider‐administered or self‐administered allowed	Provider‐administered or self‐administered allowed	Self‐administration required, unless the person is physically incapable of self‐administration (in which case practitioner administration is allowed)
2.12 Information requirements	No specific information requirements beyond consent requirements (see 1.7 consent requirements above)	No specific information requirements beyond consent requirements (see 1.7 consent requirements above)	Informed, by the first and second assessors/providers, of means available to relieve suffering, including counselling services, mental health and disability support services, community services and palliative care and offered consultations with relevant professionals who provide those services or care There must also be agreement between the first and second assessor/provider that the person has given serious consideration to means available to relieve suffering (see also 1.7 consent requirements above)	Specific information must be provided including about diagnosis, palliative care and treatment options and likely outcomes of that care and treatment (a person's understanding of this information is assessed in determining whether the person is eligible in the first assessment and consulting assessment)
2.13 Contact person	Not required	Not required	Not required	A person who is over 18 must be appointed and has legislative duties such as returning any unused medication, being contacted for feedback by the oversight body
2.14 Prospective approval	Not required	Not required	Not required	A permit (either a self‐administration or practitioner‐administration permit) must be granted by the Secretariat of the Department of Health before administration
2.15 Compliance with law	AD must be provided with reasonable knowledge, care and skill and in accordance with any applicable provincial laws, rules or standards	AD must be provided with reasonable knowledge, care and skill and in accordance with any applicable provincial laws, rules or standards	AD must be provided with reasonable knowledge, care and skill and in accordance with any applicable provincial laws, rules or standards	Coordinating medical practitioner must certify process completed in accordance with the law in the final review
2.16 Training	No mandatory training requirement	No mandatory training requirement	No mandatory training requirement	Mandatory training requirement
2.17 Oversight	Determined on a provincial basis, litigation under normal court processes available	Determined on a provincial basis, litigation under normal court processes available	Determined on a provincial basis, litigation under normal court processes available	VAD Review Board established as the oversight body; Victorian Civil and Administrative Tribunal can review some decisions; litigation under normal court processes available
2.18 Participation in VAD	Individual conscientious objection protected under s 241.2(9) of the *Criminal Code* and also governed by provincial/territorial regulator college standards Institutional objection governed by provincial law, policy and/or agreements	Individual conscientious objection protected under s 241.2(9) of the *Criminal Code* and also governed by provincial/territorial regulator college standards Institutional objection governed by provincial law, policy and/or agreements	Individual conscientious objection protected under s 241.2(9) of the *Criminal Code* and also governed by provincial/territorial regulator college standards Institutional objection governed by provincial law, policy and/or agreements	Individual conscientious objection explicitly protected in the Act Institutional objection governed by local policy

First, the legalisation processes in Canada and Victoria differ. AD was legalised federally in Canada after litigation by patients and caregivers resulted in a declaration by the Supreme Court of Canada (SCC) that the absolute prohibition on AD offended the *Canadian Charter of Rights and Freedoms* (*Charter*) and was unconstitutional (*Carter v Canada (Attorney General*) [2015] 1 SCR 331 (*Carter*)) [3, 38]. The Canadian Parliament subsequently amended the *Criminal Code* to allow AD by Bill C‐14 in June 2016 [2, 3]. AD in Canada was therefore borne out of litigation and advocacy focussed on rights by patients and caregivers such as Gloria Taylor and Kay Carter's family (and Sue Rodriguez before them) [3, 19]. This trend has continued with further changes to the Canadian *Criminal Code* made after litigation (*Truchon c. Procureur général du Canada (Truchon)*) and advocacy by patients and caregivers [4, 39, 40, 41]. The Canadian Parliament amended the *Criminal Code* through Bill C‐7 in March 2021 [4]. As a result of the litigation by two patients (Jean Truchon and Nicole Gladu) [39], the eligibility criterion requiring ‘natural death has become reasonable foreseeable’ was removed [4]. As a result of the advocacy, Bill C‐7 amended some of the procedural safeguards including introducing ‘final consent waivers’, responding to the efforts of Audrey Parker and her friends [4, 41]. Canadian law reform has also involved consultations/reports, some of which were mandated by Bill C‐14 and Bill C‐7 [42, 43, 44], as well as action by advocacy groups, civil liberties associations and others [45].

Although patient and caregiver voices were also significant in legalising AD in Victoria, including through submissions to parliamentary committees (e.g., the Inquiry into End of Life Choices Final Report) and broader advocacy efforts [46, 47], the Victorian AD framework resulted from a political process culminating in the Ministerial Advisory Panel Report [48, 49]. Emphasis throughout this Report, in parliamentary debates, and in wider political discourse in Victoria was placed on the plethora of ‘safeguards’ [49, 50, 51]. This focus on safeguards (potentially, at the expense of access) [30] is reflected in Victoria's conservative AD model, including the 127‐page *Voluntary Assisted Dying Act 2017* (Vic), containing ‘68 safeguards’ [5, 51].

The second difference is the timing of legalisation and the existence, or nonexistence, of planned implementation periods. In Canada, *Carter* was decided in 2015 [38]. Between February and June 2016, individuals could seek a constitutional exemption for AD access [3, 38]. Bill C‐14, which amended the Canadian *Criminal Code* and established an AD framework, was not enacted until June 2016 and came into effect immediately without an implementation period (though there had been 16 months between *Carter* and Bill C‐14 available for preparation) [52]. This led to a situation where practitioners and organisational decision‐makers scrambled to understand the law and establish processes [52], contributing to a regulatory ‘patchwork’. This situation was replicated with the *Truchon* decision and Bill C‐7. *Truchon* was decided in September 2019 with a 6‐month suspension on the declaration of invalidity (with further extensions to 18 months total) [39]. Individuals could seek judicial authorisation for AD access from the first extension onwards. Bill C‐7 came into effect immediately when passed in March 2021 [4]. That said, it included an exclusion of AD for persons with mental disorder as their sole underlying medical condition (MD‐SUMC) and a provision for the automatic repeal of this exclusion after 2 years (later extended for an additional year, and recently extended again) [4, 53]. This was the first instance of a period for preparing for implementation being built into the legislation in Canada.

Victoria's law was passed in 2017 and came into effect in 2019. In stark contrast to Canada (with the exception of the exclusion of AD for MD‐SUMC), there was an 18‐month implementation period between the law's passing and commencing [35], theoretically allowing time to establish procedures to facilitate AD.

Related to this is a third factor: both Australia and Canada have complex ‘regulatory’ landscapes. They are both federations, with law‐making power divided between federal, provincial/state and territorial governments. Consequently, AD is governed by a combination of federal, provincial/state and territorial law [3, 54, 55]. Professional standards issued by peak bodies (including colleges, associations or boards) are also relevant regulatory tools, given the self‐regulating nature of medical and nursing professions [56, 57]. Each system is also reliant on health practitioners and institutions to facilitate AD access. This leads to fragmentation. This is particularly so in Canada where criminal law is administered federally but health is administered on a provincial/territorial level, and to a lesser extent also in Victoria despite criminal and health law both being administered at the state level. Consequently, AD practice is influenced by many individuals and tools, which can result in gaps in regulation but also opportunities to improve AD systems through actions by different individuals and entities.

## 3 Methods

### 3.1 Design

This qualitative study is part of a wider international study examining AD regulation in Australia and two case study countries, Canada and Belgium, aiming to recommend an optimal Australian AD regulatory framework [58]. Qualitative interviews with key stakeholders in each country (patients, caregivers, health professionals, and organisational decision‐makers) aim to understand how AD operates in practice. It also aims to recommend an optimal holistic Australian AD regulatory framework, with suggestions for the case study countries too [58]. This article contributes to the wider international study by examining patients' and caregivers' experiences of AD in Australia and Canada and their suggestions for improving regulation.

This research is informed by a critical realist worldview [59]. This worldview informs the research design because we acknowledge that, although AD systems exist independently of people's experiences of them, people experience AD systems differently. This article aims to provide rich context, through reporting on qualitative, semistructured interviews, about how our sample has experienced AD and their suggestions for change.

### 3.2 Setting and Participants

As part of the wider international study outlined above, we conducted interviews with patients and caregivers in (1) Victoria, Australia; and (2) British Columbia (BC), Ontario and Nova Scotia in Canada (selected to provide geographic, experiential and regulatory diversity). In both the Australian and Canadian arms of the study, individuals were eligible to participate if they were: (1) patients seeking AD, irrespective of the outcome of the assessment and (2) caregivers supporting a patient meeting these criteria (who therefore had direct AD experience).

### 3.3 Sampling and Recruitment

In Australia and Canada, convenience sampling was used to recruit participants who contacted us in response to study advertising via advocacy group mailing lists, our website and Twitter [60]. We also used snowball sampling [61] and purposive sampling to increase diversity [60]. Purposive sampling included contacting individuals known to the research team, and advocacy groups contacting individuals known to them at our request, whose experience we sought for the research (e.g., diverse diagnoses). Recruitment ceased when the research team evaluated whether there was sufficient ‘information power’ to answer our research questions [62, 63].

### 3.4 Data Collection

Semistructured, qualitative interviews were conducted in Victoria and three Canadian provinces. Victorian interviews were conducted via Zoom (*n* = 25), telephone (*n* = 2) or in‐person (*n* = 1), between August 2021 and October 2022. Canadian interviews were conducted via Zoom (*n* = 32) between October 2021 and August 2022. B.P.W. and R.J. conducted all Australian interviews together, with one designated lead. R.J. led all Canadian interviews, with E.C. participating in 11 and JD participating in two to refine the interview guides and as part of R.J.'s research training (one interview was jointly led by R.J. and E.C. because the participant was a caregiver and health professional, relevant to the wider international study). Interviews were conducted using interview guides, developed for each jurisdiction and cohort (File S1). Examples of the questions asked were as follows:

- What would have helped to make navigating the AD system easier?
- After the process was finished, did you have any contact with anyone about how the AD system worked? Did you provide any feedback (positive or negative)?

Interviews were recorded and transcribed verbatim. Participants were able to review and amend their transcripts. Research team members debriefed after each interview and kept a reflexive journal.

All participants provided free and informed consent. Almost all participants were caregivers. They reported on their own experiences supporting patients through AD and also provided third‐party accounts of the experience of patients they were supporting to access AD. All patients whose experiences were described in interviews by caregivers who supported them had died before our interviews with their caregivers. Because of this, we were unable to seek consent from the patients whose experiences were described by caregivers.

### 3.5 Data Analysis

All transcripts, and supplementary information provided by some Victorian participants, were uploaded to NVivo (Release 1.6.1) for analysis.

Victorian interview data were analysed first. Transcripts were coded using reflexive thematic analysis (all Australian transcripts and supplementary information were double‐coded by R.J. and B.P.W.; all coding was discussed to enrich analysis) [62]. After initial coding, R.J. extracted all data relating to actions being taken by patients and caregivers to influence behaviour within the AD system, and coded this subset of data again inductively, line by line. Canadian interview data were analysed later, using codebook template analysis [64]. Analysis of Canadian data drew on the conceptual framework developed for the Victorian arm of the study but analysis occurred separately to the Australian data. Data relating to actions by Canadian patients and caregivers to influence behaviour were identified. E.C. reviewed the Canadian data to enrich analysis (in particular, by providing insight into the wider Canadian regulatory context) and sense‐check themes [62, 64].

Patients' and caregivers' suggestions for improving regulation were deductively identified as the areas of interest relevant to the research question addressed in this article. R.J. recoded each of the subsets of data from Victoria and Canada line by line using reflexive thematic analysis to identify patients' and caregivers' suggestions for how systems should respond to address identified issues that patients and caregivers were seeking to address through their actions. The subsets of Victorian and Canadian data were each initially coded separately, with coding then being reviewed as a whole to refine themes across both jurisdictions. Codes and themes were developed inductively and iteratively using reflexive thematic analysis and were discussed and refined by all authors [62]. Both latent and semantic themes are reported (i.e., sometimes suggestions were explicit and sometimes inferred).

Because of the similarities between the data from Australia and Canada, we adopted the approach of synthesising findings that resonated across both jurisdictions rather than undertaking a comparative analysis that focused on drawing out the differences.

### 3.6 Rigour and Trustworthiness

A core part of rigour and trustworthiness in reflexive thematic analysis is articulating the decisions made in data analysis [62], which we have outlined above. Core aspects of our reflexive practice included debriefing after interviews, maintaining reflexive journals for Australian and Canadian interviews and iteratively discussing interpretations of data amongst the research team.

Ethics approval for the study was obtained from the Queensland University of Technology Research Ethics Committee (2000000270) and the Dalhousie University Research Ethics Board (#2020‐5313 and #2021‐5688). This research was conducted in accordance with the requirements of our ethics approvals.

This study is reported according to the consolidated criteria for reporting qualitative research (COREQ) (File S2) [65].

## 4 Results

Sixty interviews were conducted with 67 participants (65 caregivers, 2 patients) across Victoria and Canada. In Victoria, we conducted 28 interviews with 33 participants (32 caregivers, 1 patient) about 28 patient experiences. Five interviews involved two caregivers, two interviews were conducted on separate occasions with a different caregiver about one patient experience, one participant reported two patient experiences and one caregiver's interview occurred over two dates. In Canada, we conducted 32 interviews with 34 participants (33 caregivers, 1 patient) about 33 patient experiences. One participant spoke about two patient cases and two interviews involved two caregivers. Participant characteristics are outlined in Table 2 and patient characteristics in Table 3. Overall, we were able to capture perspectives on 61 patient experiences across Australia and Canada (primarily as reported by caregivers). In all cases where a caregiver reported on a patient experience, the patient had died. Because of the difficulty recruiting eligible patients (one further patient was recruited in Australia but died before the interview), this study reports primarily on caregivers' perceptions of patient experiences, as well as their own experiences, of AD. Victorian interview duration was 56–134 min (median of 90). Canadian interview duration was 55–203 min (median of 83.5). We generated six themes (Table 4).

**Table 2:** Characteristics of participants interview participants (*n* = 67).

Characteristics	Australia (*n* = 33)	Canada (*n* = 34)	Total sample (*n* = 67)
Age (years)	Mean 56.6	Mean 58.6	Mean 57.6
20–29	1	1	2
30–39	4	2	6
40–49	7	6	13
50–59	3	8	11
60–69	13	8	21
70–79	4	7	11
80–89	1	2	3
Gender			
Female	26	25	51
Male	7	9	16
Relationship to patient	*	*	*
Child (including stepchild, child in‐law)	17	17	34
Spouse (including de facto partner)	10	12	22
Parent	3	2	5
Close friend	1	2	3
Niece/nephew	0	1	1
Sibling	2	0	2
Self	1	1	2
	* One participant reported two cases, so this category is calculated by using the total number of relationships (*n* = 34)	*One participant reported two cases, so this category is calculated by using the total number of relationships (*n* = 35)	*Two participants reported two cases, so this category is calculated using the total number of relationships (*n* = 69)

**Table 3:** Characteristics of patients described in interviews (*n* = 61).

Characteristics	Australia (*n* = 28)	Canada (*n* = 33)	Total sample (*n* = 61)
Age (years)	Mean 70.8	Mean 70.8	Mean 70.8
20–29	1	0	1
30–39	1	2	3
40–49	0	2	2
50–59	3	1	4
60–69	7	6	13
70–79	8	12	20
80–89	6	9	15
90–99	2	1	3
Gender			
Female	13	18	31
Male	15	15	30
Illness type			
Cancer	18	20	38
Neurological	9	6	15
Other (e.g., COPD, heart disease)	1	7	8
State/province			
Victoria, Australia	33	N/A	33
Ontario, Canada	N/A	14	14
British Columbia, Canada	N/A	11	11
Nova Scotia, Canada	N/A	6	6
Other, Canada	N/A	2*	2
		*The participants who reported on patient cases relating to other provinces were either located in one of the target provinces or also reported on a patient case that occurred in one of the target provinces, hence the inclusion in our sample	
Eligibility for AD			
Assessed as eligible	24	30	54
Patient died via self‐administered AD substance	19	0	19
Patient died via practitioner‐administered AD substance	3	29	32
Patient died but did not take AD substance (natural death)	1	1	2
Patient waiting to take AD substance	1	0	1
Patient died before eligibility assessment completed	3	2	5
Patient assessed as ineligible and died	1	0	1
Currently in assessment process	0	1	1
Timing of death (or engagement with the process)			
2016	N/A	1	1
2017	N/A	5	5
2018	N/A	10	10
2019	4	3	7
2020	9	5	14
2021	15	9	24
Education level			
Postgraduate university	4	12	16
Undergraduate university (including diploma‐level, or incomplete qualification)	8	10	18
Community college	0	3	3
High school	9	5	14
Some high school	7	3	10

**Table 4:** Overview of suggestions from patients and caregivers and how relevant stakeholders should act to address suggestions.

Theme	Issue identified by patients and caregivers	Suggestion by patients and caregivers to address issue	Aspects of suggestions by patients and caregivers to address issue	Examples of how key stakeholders should act on suggestions to address issues
Theme 4.1	Lack of knowledge about AD as a lawful option and about the process of accessing AD	Improved content and dissemination of information about AD	1. Remove legal prohibition on discussing AD with patients (Victoria, Australia)	Victorian Parliament should amend AD legislation to remove the prohibition on raising AD with patients Regulatory bodies and health and medical groups should issue guidelines saying that there is a duty to inform patients about AD
			2. Better integrate AD into end‐of‐life care, and particularly palliative care, and provide information through these channels	Policymakers and government officials should introduce policies highlighting the importance of integrating AD into broader end‐of‐life care Peak bodies and regulatory colleges should issue guidance or professional standards about information provision and the relationship between AD and other areas of end‐of‐life care Healthcare institutions should provide guidance about information provision about AD via institutional policies Healthcare professionals, particularly palliative care professionals, should reflect on their own practice and how it relates to AD Universities and other training providers should incorporate AD into curricula
			3. Require objecting health practitioners to provide information about AD	Parliaments should introduce amendments to legislation to require information provision Peak bodies and regulatory colleges should issue guidance or professional standards Healthcare institutions should provide guidance to objecting staff, including via institutional policies
			4. More end‐of‐life training and education for doctors and other healthcare professionals	Peak bodies and regulatory colleges should provide incentives for undertaking training (e.g., accrediting training courses for continuing professional development) Healthcare institutions should facilitate training courses, provide study leave and so forth Healthcare professionals should seek out training and education Universities and other training providers should incorporate AD into curricula
			5. Information strategies about AD, including information for diverse population groups	Policymakers and government officials should develop and distribute information and resources in consultation and partnership with diverse population groups Peak bodies and regulatory colleges should issue guidance or professional standards about engaging with diverse population groups Healthcare institutions should provide guidance via institutional policies, ensure appropriate staffing to support diverse patient groups and facilitate interdisciplinary teams Healthcare professionals should sensitively, where appropriate and in accordance with good clinical practice, discuss AD with patients and involve appropriate members of interdisciplinary teams (e.g., interpreters, speech pathologists, social workers, cultural liaison officers) Universities and other training providers should provide training about care considerations for diverse population groups
			6. Ensure patient‐ and family‐facing resources are accessible, clear and person‐centred (and include experiential and emotional aspects of AD in addition to legal considerations)	Policymakers and government officials should revise information and resources to ensure they are not overly legalistic Healthcare institutions should share resources (e.g., display or provide resources onsite) Healthcare professionals should share resources about AD, where clinically appropriate Advocacy organisations should continue to share information about AD experiences
			7. Accurate media reporting about AD	Media should ensure reporting about AD is accurate
			8. Increase cultural capacity to have more nuanced discussions about AD	Policymakers and government officials should develop information and resources about death and dying (including information and resources for patients and caregivers pre‐AD and post‐AD) and be mindful of chosen language to describe AD Media should ensure clear and accurate reporting about AD Advocacy groups and other organisations should facilitate space for discussion about the nuanced experiences of AD Grief and bereavement organisations should support discussion about complexities in AD experiences
			9. Destigmatise and normalise AD, including via the strategies above as well as: − Considering the language used for AD and other types of medically assisted deaths − Improving death literacy and normalising death and dying in general	Peak bodies and regulatory colleges should issue guidance or professional standards using appropriate language Healthcare institutions should provide guidance via institutional policies about appropriate language Healthcare professionals should be mindful of using appropriate and accurate language when discussing AD with patients, and incorporate discussions about AD into end‐of‐life care Universities and other training providers should provide training about end‐of‐life care, including death and dying more broadly Media should use appropriate and accurate language to discuss AD Advocacy groups and other organisations should share information about death and dying Grief and bereavement organisations should support discussion about complexities in AD experiences
Theme 4.2	Lack of policies and procedures relating to the provision of AD (particularly in relation to institutional participation in AD)	Proactively develop policies and procedures about AD provision	1. Proactively develop transparent institutional policies around participation in AD	Healthcare institutions should develop policies around participation, and advertise policies clearly to staff, patients/residents and prospective patients/residents
			2. Develop policies to facilitate organ donation that maximise patient autonomy	Policymakers and government officials should develop information and resources about organ donation and AD Healthcare institutions should develop institutional policies to facilitate organ donation after AD Healthcare professionals should discuss (sensitively and where clinically appropriate) organ donation after AD Organ donation organisations should develop policies to facilitate donation after AD
Theme 4.3	Harms associated with institutional objection	Address institutional objection via top‐down action	1. Attach conditions to public funding provided to hospitals that require hospitals to facilitate AD	Parliaments should introduce legislation to require hospitals to facilitate AD if publicly funded
			2. Develop expectations for institutional responses from the outset (that are directed by government decision‐makers)	Policymakers and government officials should proactively set expectations for institutional participation in AD
			3. Monitor policies to ensure they are not creating additional and unnecessary barriers to access	Policymakers and government officials should listen and respond to complaints about institutional handling of AD Peak bodies and regulatory colleges should issue guidance or professional standards Health professionals should report concerns about institutional policies that are overly restrictive or hamper access
			4. Have a dedicated, standalone facility for AD	Policymakers and government officials should consider creating dedicated, standalone AD facilities Nongovernmental organisations or healthcare institutions should consider establishing dedicated, standalone facilities for AD
			5. Set aside a room to facilitate AD within objecting institutions	Healthcare institutions should set aside areas to facilitate AD on‐site
Theme 4.4	Absence of resources relating to grief and AD	Proactively develop grief resources and peer support mechanisms	1. Proactively develop grief resources, including for particular cohorts (e.g., children, diverse population groups)	Policymakers and government officials should proactively develop grief resources Healthcare institutions should develop grief resources or promote and display existing grief resources Health professionals should reflect on the grief and bereavement support they will provide after participating in AD Advocacy groups should develop or promote existing grief resources Grief and bereavement‐specific organisations should develop grief resources that include AD‐specific considerations
			2. Proactively develop peer support mechanisms	Policymakers and government officials should develop peer support mechanisms Advocacy groups should develop peer support mechanisms Grief and bereavement‐specific organisations should establish peer support mechanisms
Theme 4.5	Experiences of legal barriers	Amend laws to address legal barriers	1. Proactively amend the law to address legal barriers, including overly restrictive eligibility criteria, unnecessary procedural safeguards and other legal barriers	Parliament should amend laws to address legal barriers
Theme 4.6	Lack of established feedback mechanisms or unwillingness to engage with/inattention to patient and family feedback and voices	Engage with and act on patient and caregiver feedback	1. Routinely and proactively collect and engage with and act on patient and caregiver feedback	Policymakers and government officials should establish feedback collection mechanisms (e.g., the VAD Review Board in Victoria) Healthcare institutions should routinely seek feedback from patients and caregivers about experiences and improve their AD systems Health professionals should routinely seek feedback from patients and caregivers about their experiences Advocacy organisations should collect information from patients and caregivers about perspectives on AD Researchers should engage patients and caregivers in research about AD
			2. Share information amongst providers of feedback provided by patients and caregivers (i.e., avoid siloing)	Policymakers and government officials should establish communities of practice for providers Peak bodies and regulatory colleges should support communities of practice Health professionals should debrief with colleagues about experiences and feedback Training providers should incorporate common issues or feedback into training programmes
			3. Include patients and caregivers in system design and evaluation (including research)	Policymakers and government officials should establish feedback collection mechanisms (e.g., the VAD Review Board in Victoria), and engage consumer representatives Healthcare institutions should seek feedback from patients and caregivers about experiences and improve their AD systems Health professionals should seek feedback from patients and caregivers about their experiences Advocacy organisations should collect information from patients and caregivers about perspectives on AD Researchers should engage patients and caregivers in research
			4. Make efforts to ensure diversity in representation (e.g., Indigenous people)	Policymakers and government officials should ensure diversity in representation when consulting about AD or developing AD policies in partnership with consumers or institutions Healthcare institutions should ensure diversity in representation when developing policies or guidelines for AD within the institution Researchers should seek diverse perspectives for research

### 4.1 Improved Content and Dissemination of Information About AD

Most participants highlighted the need for greater information about AD as a *lawful option* because there ‘really isn't a great awareness of it’ (Caregiver interview 3, Ontario). Even in Canada, where AD was legalised earlier, participants described this as a problem and commented on the central role of not‐for‐profit organisations in providing AD information.

Participants also wanted more information on the *process* of accessing AD due to the ‘lack of generalised information about that process that exists’ (Caregiver interview 12, Nova Scotia), with one participant describing trying to find information as like ‘putting pieces of a puzzle together’ (Caregiver interview 11, Ontario).

These concerns were reported particularly by participants with AD experiences in Victoria, Ontario and Nova Scotia. In BC, information was described as being available, with one participant commenting that, for a general audience, ‘the information on the BC Ministry Healthcare page was super‐clear’ (Caregiver interview 27, BC/Canada) but raised concerns about accessibility for diverse groups.

Patients and caregivers undertook efforts themselves to share information to bridge this gap:

…I've made it my mission to share as much as I can about AD…

(Caregiver interview 16, Victoria/Australia)

Participants had various suggestions about how AD systems could better disseminate information about AD.

A key suggestion from Victorian participants related to the legal prohibition preventing healthcare practitioners from raising AD as an option unless the patient raises it first (Table 1). Participants thought this should be repealed so information could be provided openly within healthcare consultations:

…it's ridiculous that the doctors can't proactively mention this…borderline negligent…

(Caregiver interview 17, Victoria/Australia)

Some participants also commented that information about AD should be provided as part of end‐of‐life care, including as part of palliative care:

…it's up to the palliative care doctors to…make that a part of their role…when you're being palliative…you're at the end of life, and these options should be brought forward.

(Caregiver interview 3, Ontario/Canada)

Further, participants suggested all doctors, including those who conscientiously object, should be required to provide information. Additionally, participants highlighted a need for more end‐of‐life training for doctors and other healthcare professionals so they understand the AD process and can appropriately provide information.

Information strategies should ensure accessibility of information to people from diverse backgrounds, like older people who may not be Internet literate or those from culturally and linguistically diverse backgrounds. Suggestions for accessibility also related to the *content* of information needing to be less legalistic and more person‐oriented:

…they'd [Victorian government] written the protocols and the framework and the guidelines to meet the legislation…They hadn't written the policies, the protocols and the guidelines to meet the requirements of someone accessing the VAD system…it was just that disjunct between the system being legal and ensuring that there were no errors at all from a legal perspective…it wasn't a person‐centred process…

(Caregiver interview 21, Victoria/Australia)

Additionally, participants highlighted the importance of resources emphasising the emotional and experiential aspects of AD, including drawing on stories of individuals with lived experience:

…I was desperately looking for examples … of other people who had gone through this…to get more of an idea of what the hell to expect…we couldn't find anything. It was all policy driven and it wasn't really about the personal experience…and now I'm across the Go Gentle [patient information and advocacy organisation] page where they've got a whole…repository…

(Caregiver interview 24, Victoria/Australia)

One participant commented on inaccurate media representation of AD, what is involved and a need for more accurate information dissemination:

…it was spun in the media…I'd really like to see us highlight, so that there's a greater public understanding of what the process is, that it's not an easy process.

(Caregiver interview 18, BC/Canada)

Linked to this was a feeling by participants that they were unable to discuss the complexities of their experience of AD because of opposition to AD and the discomfort that exists, and suggested a need to increase cultural capacity to have more nuanced discussions and information provision about AD:

I don't think there's much of a platform in public discourse right now for discussing the complexities of the experience…because of folks getting this legislation across the line there's not much room to say, well, this part of it was really traumatic…

(Caregiver interview 20, Victoria/Australia)

Participants identified that part of improving information provision about AD involves destigmatising AD. Participants perceived that strategies for destigmatising AD should include those outlined above but acknowledged the specific need and effect of those strategies to reduce stigma. One participant commented that healthcare professionals should be allowed to initiate AD discussions because:

…It would be an important way to actually sort of normalise VAD…the reason that it's [AD] taboo is because it's treated differently.

(Caregiver interview 12, Victoria/Australia)

Additionally, participants felt that integrating AD into other end‐of‐life choices, like palliative care, was needed to destigmatise AD:

…we need to…destigmatise VAD or MAiD and make it just one of the many end‐of‐life choices you have that are all medically assisted…

(Caregiver interview 9, Ontario/Canada)

Participants also commented on the need to increase death literacy and normalise death and dying in general.

### 4.2 Proactively Develop Policies and Procedures About AD Provision

Participants reported a need for proactive development of policies and procedures about AD provision. A key area of concern for participants, particularly those in Victoria, Ontario and BC, was institutional policies.

Some participants experienced interactions with institutions that had not developed policies relating to AD, or whose policies were unclear, and sought to address these issues:

I'm hoping that people's experiences now [relating to lack of policies/procedures] will be nothing like what ours were…I participated in some of those media opportunities…to try to get people to be aware…

(Caregiver interview 21, Victoria/Australia)

Sometimes a lack of policies or procedures was perceived to stem from the institution's objection to AD, but other times as the result of institutional disorganisation:

They were unorganised, both in terms of the technical procedures and what you had to go through, but also and more important culturally equipped to deal with it. Their staff didn't know how to react. They had no protocol…

(Caregiver interview 11, Ontario/Canada)

Participants suggested institutions should *proactively* develop policies and procedures to handle AD on site:

Healthcare systems don't respond well when you throw them a curve or you throw them something new…I'm hoping that someone in my mum's situation today would have a better experience…because it's become more part of procedure.

(Caregiver interview 11, Ontario/Canada)

Implicit in participants' comments was a need for transparency about what aspects of AD institutions are willing to be involved so patients and caregivers can make choices about end‐of‐life care. One participant was ‘gobsmacked’ because the CEO of the institution the patient sought AD in wrote to the caregivers to the effect that facilitating AD was ‘something that we're glad to be able to do, albeit quietly’ (Caregiver interview 16, Victoria/Australia).

Beyond institutional objection, one Canadian participant suggested developing policies and procedures for organ donation that maximise a patient's ability to exercise autonomy at the end of life.

### 4.3 Address Institutional Objection via Top‐Down Action

While some institutions failed to develop policies about AD (Theme 4.2), others developed explicit policies against AD or some aspects of it. Many participants perceived this as harmful. This issue was particularly raised in Victoria, BC and Ontario.

Patients and caregivers attempted to overcome the impacts of institutional objections to AD through actions such as advocating to the institution to allow aspects of the AD process to occur onsite:

We came forward, worked with…the British Columbia Civil Liberties Association. The rabbi of the synagogue wrote a letter. I wrote a letter. Mama wrote a letter.

(Caregiver interview 27, BC/Canada)

While sometimes participants reported effectively mediating the negative impacts of institutional objection, other times, their efforts to overcome these impacts were unsuccessful:

…I don't think that they're going to respond to heartfelt pleas…

(Caregiver interview 27, BC/Canada)

Participants had several suggestions about how AD systems could better respond to address the harmful impacts of institutional objection. A key suggestion was top‐down action to influence objecting institutions' positions, like attaching conditions to public funding:

It has to come from the province and say ‘…these are things you're required to do. And if you don't want to, you don't have to take any of the provincial funding…’

(Caregiver interview 27, BC/Canada)

One participant also commented on the effectiveness of organisational decision‐makers setting the tone from the outset:

They just came down hard on that [institutional objection] right from the very beginning…my understanding is that [other province] has very advanced and very supportive programs for MAID.

(Caregiver interview 9, Ontario/Canada)

Participants also spoke of policies or procedures being implemented by institutions that were perceived to act as roadblocks:

…that was one of these other brick walls they tried to throw up, is ‘No, we won't accept her form. You have to use our form and our lawyers have to sign it’…they just kept coming up with nonsense…

(Caregiver interview 23, Ontario/Canada)

A policy can purport to facilitate AD but, by adding additional and unnecessary barriers, can hamper access to AD in practice. Therefore, implicit in participants' comments was a need to ensure that policies do not add unnecessary barriers.

Participants also suggested two other strategies that could be implemented at a government or organisational level, which could help mediate some of the harmful impacts of institutional objection.

The first was the creation of a dedicated, standalone facility to support AD provision (an approach which exists in Canada, e.g., MAiDHouse in Ontario/Canada):

…should we have a facility…somewhere proximate to services…

(Caregiver interview 17, Victoria/Australia)

The second was that objecting institutions should have ‘a room set aside’ (Caregiver interview 21, Victoria/Australia) to facilitate AD. This strategy has been implemented in Nova Scotia and one participant commented positively on this approach:

…one Catholic hospital and nursing home…have since got around that by designating a part of the hospital…the patients are transported to this one room…

(Caregiver interview 13, Nova Scotia/Canada)

### 4.4 Proactively Develop Grief Resources and Peer Support Mechanisms

Participants across all jurisdictions highlighted a need for proactive development of grief resources and peer support mechanisms. An absence of grief resources resulted in participants developing those resources for others:

…there were no resources…I've since written two children's books about MAID…

(Caregiver interview 5, BC/Canada)

Participants commented on the need for resources relating to grief, including for particular cohorts, such as children or diverse population groups:

I went hunting…[for resources about talking to children about AD]…that would be fantastic to have.

(Caregiver interview 17, Victoria/Australia)

Participants also suggested that grief‐ and bereavement‐specific organisations should be aware of the unique nature of AD grief and proactively develop resources:

I reached out to the Australian Bereavement Society and…said ‘…it's something you need to be aware of or to be thinking of…it's not ordinary grief, there is something quite unique about it.’

(Caregiver interview 21, Victoria/Australia)

The importance of peer support mechanisms was also highlighted by several participants, some of whom had offered their peer support to others in informal or formal peer support roles. It was implicit in participants' comments that peer support networks should be proactively developed:

I remember thinking, I wish I could talk to other parents who are going through what I'm going through and there was no‐one.

(Caregiver interview 7, Ontario/Canada)

### 4.5 Amend Laws to Address Legal Barriers

Patients and caregivers experienced legal barriers relating to eligibility criteria and procedural safeguards perceived as overly restrictive. This finding was reported in all jurisdictions, though the precise nature of legal barriers differed.

Participants also commented on the cumulative impact of procedural safeguards:

…having a flexible, pragmatic mindset in the legislation … rather than a kind of paradigm that says…we want to make it…as hard as possible for anyone else to interfere in the process…they've got that balance unduly skewed towards the latter.

(Caregiver interview 17, Victoria/Australia)

Participants sought to overcome legal barriers themselves. For example, one participant supported a patient who could not access AD due to not being an Australian permanent resident, as required by Victoria's law (Table 1), and sought to change this:

I did the research and wrote this paper…I've been pushing and pushing…if I could get this monkey off my back…I could get on with the rest of my life…

(Caregiver interview 5, Victoria/Australia)

A Canadian participant spoke of the requirement for final consent in all AD cases, which existed before March 2021 (see Table 1), which prompted the patient to (successfully) undertake coordinated efforts to change the law.

The obvious solution identified by participants is to change laws in ways that target identified barriers, for example, repealing or changing the law which effectively precludes telehealth from being used to discuss AD in Victoria (Table 1):

…I've written to a couple of federal MPs about this and the relevance of this law…[the inability to use telehealth] was hugely impacting…

(Caregiver interview 6, Victoria/Australia)

Importantly, a key implication of participants' comments was that laws should be changed proactively, rather than reactively to court decisions that require affected individuals to litigate, often at great personal cost:

That's what we've had to do in Canada, because the politicians do listen to the courts. So unfortunately, often posthumously, families have to come forward and document and show the tremendous stress financially, emotionally, physically and mentally that the dying person was subjected to because of the flaws in the law.

(Caregiver interview 9, Ontario/Canada)

Participants also highlighted a need to recognise the importance of law evolving over time, in accordance with evidence:

…you can't just create it [law] once and think you're done … starting off fairly narrow and broadening as people see fit and as you learn from other countries…that's the only way…and data…that's how you understand things like this…by science.

(Caregiver interview 12, Nova Scotia/Canada)

### 4.6 Engage With and Act on Patient and Caregiver Feedback About Experiences

Participants commented on the importance of engaging with and acting on patients' and caregivers' feedback about their experiences because patients and caregivers are the most affected when things go wrong:

I think the important part is that for us you only get to do it once. There's no do‐overs.

(Caregiver interview 9, Ontario/Canada)

This theme overlaps with and overarches the other five themes: part of engaging with and acting on feedback is taking on board suggestions, such as those outlined in the other five themes.

Participants had several suggestions for how systems could be more responsive to their feedback. Some Canadian participants perceived their feedback was not proactively sought and commented on the importance of this:

…it would have been nice to have had an organisation…to have reached out to offer support. And also to gather data…what you're doing now, they could have got all this information from me…maybe it could have helped other people…

(Caregiver interview 30, BC/Canada)

Because the Victorian AD model involves the collection of feedback by the VAD Review Board after every AD case, this was not reported in Victoria.

Some participants provided feedback about their experience directly to the clinical team and commented on the importance of using and sharing this information to shape practice. One participant spoke of an instance where a person's assisted death was impacted because they were dehydrated, which resulted in a very difficult administration and suffering for the patient and caregivers:

…I've been urging the doctors, the clinicians, advisory council, especially at Dying With Dignity [patient education and advocacy group], to please create a network where you share potential things that can go wrong…prepare a document for physicians, little flags, you know have you done this, have you done that, just to make sure it can go ahead.

(Caregiver interview 9, Ontario/Canada)

Participants also highlighted the importance of including patients and caregivers in the evaluation of AD systems, including through parliamentary processes and in research:

…I said ‘…You [parliamentary committee] need outside influence because all you're doing is chewing it up and spitting it out in a different way, same end result.’

(Caregiver interview 24, Ontario/Canada)

…it was really great that the researchers wanted to include us…we have equal weight in the discussion…

(Caregiver interview 12, Nova Scotia/Canada)

The lack of diversity in AD advocacy was raised as an issue, with the need to engage people from diverse populations (e.g., Indigenous people) in system evaluation highlighted.

## 5 Discussion

### 5.1 Overview

Six improvements were suggested by participants to address issues identified in their AD experience:

1. Improved content and dissemination of information about AD;
2. Proactively develop policies and procedures about AD provision;
3. Address institutional objection via top‐down action;
4. Proactively develop grief resources and peer support mechanisms;
5. Amend laws to address legal barriers;
6. Engage with and act on patient and caregiver feedback about experiences.

Despite substantive differences between Victorian and Canadian AD laws, these suggestions were common to both jurisdictions, though with some nuance. Table 4 summarises these suggestions. The key suggestions have three main functions: building awareness about AD, overcoming barriers to access, and providing more support to patients and families (both to improve AD experiences and grief and bereavement).

### 5.2 Significance of Findings

Literature in Canada and Australia identifies issues with AD regulatory systems, such as difficulties in finding information about AD and lack of process clarity [20, 21, 22, 28, 29, 30], the impacts of institutional objection [25, 26, 27] and other legal barriers [20, 21, 22, 23, 24]. The specific nature of AD grief is also highlighted [66, 67]. Some of these issues are also reflected in scholarship on AD from other countries. For example, a recent systematic review by Gamondi et al. (reporting on studies from the Netherlands, Switzerland, the United States and Canada) highlighted the lack of knowledge of AD laws and processes amongst some patients and families, and the particular nature of AD grief and bereavement [68]. Gamondi et al. called for further research into how to support caregiver needs in relation to the different models of AD [68]. Our findings resonate with these studies but are novel because we identify concrete suggestions from patients and caregivers *themselves* for how other actors in AD systems could act to address these issues. Patients' and caregivers' position as ‘users’ of AD systems uniquely places them to identify suboptimal aspects of AD systems and suggest improvements. We might reasonably expect that they provide feedback about their experiences to illuminate these issues and suggestions, but they should not be expected to (and often cannot) take other actions to influence AD systems, such as altering laws or policies. This is particularly so given how unwell people seeking AD are [13, 17], and the grief that caregivers may experience [66, 67]. It is incumbent on others to listen to feedback and respond accordingly.

Our study focuses on AD in Canada and Australia but our findings are significant in the context of global AD regulatory discourse. While Canada and Australia have very different AD regulatory systems, it was striking that the suggestions by patients and caregivers to improve AD systems resonated strongly across both jurisdictions, with some minor variation. In light of this, and given that issues identified in Canadian and Australian AD systems are similar to those identified globally [68], suggestions from patients and caregivers may be helpful in improving AD systems in other jurisdictions. A particularly interesting aspect of our findings is that only a small number of suggestions from patients and caregivers rely solely on government or parliamentary action to amend the law (Table 4). While some key legal barriers can only be addressed through law reform, which may be protracted and lengthy, our findings highlight that AD systems can be improved through the actions of a variety of actors. Our findings also highlight the power (and responsibility) of nongovernment actors, such as healthcare institutions and healthcare professionals, in acting to improve AD systems.

### 5.3 Comparative Responses in Canada and Australia

Australia and Canada have responded differently to identified issues. Understanding these comparative responses and articulating some of the factors that have made Canada more responsive to patient and caregiver suggestions than Australia is of utility for jurisdictions globally in reviewing legalising, implementing or reviewing AD systems. In 2021, Canada made changes to its law through Bill C‐7 [4]. Some changes, like removing the requirement for natural death to be ‘reasonably foreseeable’ from eligibility criteria, were required by court decisions resulting from litigation by patients and caregivers [39, 40, 52]. The key case was *Truchon*, litigated by two patients, Jean Truchon and Gloria Taylor, whose natural deaths were not reasonably foreseeable [39]. But litigation may come at great personal cost, and systems should respond before litigation becomes necessary. While actions by patients and caregivers have influenced some institutions' positions [25], other institutions have not been so responsive. This is highlighted by the recent case of Sam O'Neill who was forcibly transferred from an objecting institution [69]. Patients' families will be involved in pending litigation about the BC policy, which effectively allows faith‐based publicly funded institutions to refuse to allow AD [70], but if governments or organisational decision‐makers had responded proactively, burdensome litigation may not be necessary.

A more positive example of responsiveness in Canada relates to Audrey Parker's advocacy. Audrey chose to die by AD earlier than she wanted because her cancer was spreading and she feared she would lose capacity and would be unable to meet the final consent requirement [41, 71]. Audrey's (and her friends' advocacy) resulted in an exception to this requirement being introduced by Bill C‐7, the ‘final consent waiver’ (Table 1) [4]. While perhaps less burdensome than undertaking litigation, this concerted advocacy nonetheless required significant effort. Finally, the Canadian government, parliamentary committees and independent expert groups have undertaken extensive, proactive consultations/reviews into AD (some of which were mandated by Bill C‐14 and Bill C‐7), and have considered submissions and evidence from patients and caregivers. These reviews have considered legislative reforms to eligibility criteria and procedural safeguards, including about AD for mature minors [42], for persons with mental disorder as the sole underlying medical condition [42, 43] and to allow advance requests for AD [42]. While some consultations/reviews have been legislatively mandated, others were more proactive (e.g., the Government consultation resulting in the What We Heard Report, which contributed to changes in the law, such as reducing the required witnesses from two to one) [44].

Australian responses to address identified issues have been less positive. The former Victorian Premier publicly responded to evidence of barriers to AD access indicating no intention to change the law [72]. The Victorian government has also confirmed the (only) mandated legislative review of Victoria's law [35], occurring currently, ‘will not consider any changes to the legislation.’ [73] This means identified issues will continue to exist in Victoria and the burden of taking action to overcome those issues may continue to fall to patients and caregivers, as well as doctors, other health professionals and advocacy groups.

There may be several explanations for the relative success of Canadian as opposed to Australian responses. A simple reason may be that Canada has had 3 more years to grapple with AD and respond to identified issues. Another reason may be that Canada's pathway to AD legalisation, which was patient‐focused and rights‐based, set the tone for refining law in line with patient rights. This is highlighted by the case of *Carter*, in which the SCC accepted that an absolute prohibition on physician‐assisted dying was unconstitutional based on s 7 of the *Charter* (the right not to be deprived of life, liberty and security of the person except in accordance with the principles of fundamental justice) [38]. As prominent regulatory scholars Hancher and Moran suggest, whoever ‘commands the necessary resources at the historical moment when regulation is initiated has a good chance of exercising a continuing dominant influence.’ [74] In the context of the legalisation of AD in Canada, patients, caregivers and *Charter* rights were integral contributors to that historical moment, alongside litigation teams and civil liberties groups [45]. The *Charter* itself has provided patients and caregivers a sword to wield to initiate change, and the threat of successful litigation grounded in *Charter* rights looms over parliamentarians and law‐makers and encourages and shapes change [54]. Conversely in Australia, the AD process has been highly political and followed more than 40 unsuccessful legislative efforts [75], meaning that some aspects of the current regulatory system are the result of political compromise [76]. A final reason may be that the nature of Canadian regulation, and particularly the federal–provincial AD regulatory patchwork, has left space and opportunity for patients and caregivers to use their AD experiences to alter the operation of AD in practice, which, combined with the underlying political will to refine AD regulation (and the Liberal Government, in power since 2015), has resulted in progressive change. In Australia, the very prescriptive nature of AD models and a lack of political will to review the legislation may unfortunately leave less room for patient‐ and caregiver‐driven legal change.

### 5.4 Strengths and Limitations

This study expands knowledge by providing concrete suggestions from patients and caregivers to improve AD systems (both what needs to change and how it can/should be changed by those with the power to make change). These findings are useful for jurisdictions reviewing their systems and jurisdictions considering or implementing AD systems.

A strength is that we report on the experiences of 67 participants across two countries, with experiences from 2016 to 2022. A limitation is that only two participants were patients. Recruiting patients was difficult due to how unwell patients seeking AD are. Further research with patients is needed and may provide new or different insights. However, caregivers in this study reported on patients' perspectives and suggestions (as well as their own) and we were able to capture patient perspectives this way. Caregivers are reliable third‐party reporters for patient experiences relating to quality of care, though their perceptions may differ due to factors such as grief [77].

Only one Canadian interview related to an experience where the person's natural death was not reasonably foreseeable. Further research with this cohort and other diverse experiences, such as ineligible persons, are needed. Research with patients and caregivers in other Australian and Canadian jurisdictions (or in other countries around the world) with different structures for AD would enrich the understanding of suggestions for improvement.

Our participants supported AD in principle and, despite the diversity in experiences, all caregiver participants supported their family member's decision to seek AD (this was sometimes explicitly raised by participants or was implicit in their comments). Additionally, our recruitment strategy included recruitment through advocacy organisations, which meant that some participants had direct connections with advocacy groups. Additionally, this may have resulted in our study attracting participants with experience in trying to influence regulation. Further research with individuals who are unsupportive of or ambivalent about AD may provide new or different insights. For example, some who are opposed to or ambivalent about AD or their family member's choice to access AD might have different suggestions about how to improve AD regulation.

Although our findings may be useful for jurisdictions reviewing or implementing AD systems, our findings are not intended to be generalisable because of our qualitative design.

## 6 Conclusion

It is imperative that health systems monitor and respond to suggestions from patients and caregivers with firsthand experience of AD systems. As users of AD systems, they are uniquely placed to identify issues and make suggestions to improve these issues. We outlined six suggestions made by patients and caregivers with experiences of AD in Australia and Canada, and we argue these should be considered in reviewing existing AD systems and designing and implementing AD systems in the future. To date, Canada has responded comparatively well to feedback and suggestions by patients and caregivers for improvement, including through government consultations and Bill C‐7 changes, and parliamentary committee and expert reviews that substantively consider improvements to the law. In Victoria, the response has been less positive, which may result in the identified issues and access barriers continuing to exist in Victoria and affecting the experience of patients and caregivers.

## Author Contributions

**Ruthie Jeanneret:** conceptualisation, methodology, formal analysis, writing–review and editing, writing–original draft, investigation. **Eliana Close:** conceptualisation, investigation, methodology, writing–original draft, writing–review and editing, formal analysis, supervision. **Lindy Willmott:** conceptualisation, investigation, writing–original draft, methodology, writing–review and editing, supervision. **Jocelyn Downie:** writing–original draft, writing–review and editing, investigation, supervision. **Ben P**. **White**: conceptualisation, investigation, writing‐original draft, methodology, writing‐review and editing, supervision.

## Disclosure

The funder only provided funding and did not participate in or influence this research.

## Ethics Statement

Ethics approval for both the Australian and Canadian arms of the study was obtained from Queensland University of Technology (Reference #2000000270) and Dalhousie University (References #2020‐5313 and #2021‐5688). We conducted the research in accordance with our ethical approvals, and with the Australian National Health and Medical Research Council's National Statement on Ethical Conduct in Human Research 2007 (updated 2018) and the Canadian Tri‐Council Policy Statement: Ethical Conduct for Research Involving Humans TCPS 2 (2018) (updated in 2022).

## Consent

All persons interviewed provided free and informed consent to participate in this research.

## Conflicts of Interest

Ben P. White and Lindy Willmott were engaged by the Victorian, Western Australian and Queensland state governments in Australia to develop the legislatively mandated training for providers of AD. Ruthie Jeanneret and Eliana Close were employed on these projects. Lindy Willmott is a member of the oversight body for AD in Queensland, the Voluntary Assisted Dying Review Board. Ben P. White is a member of the Queensland Civil and Administrative Tribunal, which has jurisdiction over some matters related to AD. Jocelyn Downie was involved in initiatives contributing to MAiD law reform in Canada, including the Royal Society of Canada Expert Panel: End‐of‐Life Decision Making; the plaintiff's pro bono legal team in *Carter v Canada (Attorney General)* [2015] 1 SCR 331; the Provincial‐Territorial Expert Advisory Group on Physician‐Assisted Dying; and the Council of Canadian Academies Expert Panel on MAiD. Jocelyn Downie was also involved in developing a module for the federal MAiD National Curriculum (developed by the Canadian Association of MAiD Assessors and Providers and funded by Health Canada) and was a member of the MAID Practice Standard Task Group. Jocelyn Downie is also on the Advisory Board for the Completed Life Initiative in the United States. All views expressed in this article are those of the authors and not the organisations they are affiliated with.

## Supporting information

Supporting information.

Supporting information.

## Data Availability

The interview guides used in the study are available in the Supporting Information. Other data are unavailable due to confidentiality undertakings given to research participants as required by the study's ethical approvals. Requests to discuss this should be directed to the corresponding author.
