# Supplementary material for: Patients' and Caregivers' Suggestions for Improving Assisted Dying Regulation: A Qualitative Study in Australia and Canada
Source: Health Expect. 2024 Jun 19;27(3):e14107. doi: 10.1111/hex.14107 (PMC11187863; doi:10.1111/hex.14107)
Supplement: Supplementary file 1 — Supporting information. [file HEX-27-e14107-s002.docx]

**Patients’ and caregivers’ suggestions for improving assisted dying regulation: A qualitative study in Australia and Canada**

**Supplemental File 1: Interview guides**

**Australia – patients and caregivers**

[This semi-structured interview guide was written for both patients and family caregivers. For generic issues such as the process of seeking voluntary assisted dying, it was written in language for patients but was adapted in the interviews with family caregivers to be expressed as seeking their perception of the patient’s experience of these matters. However, some sections needed to be specifically directed to patients or family caregivers (e.g. after death experiences) and this is indicated by the headings below. Where relevant experiences outside the interview guide were raised, they were also explored.]

**Preliminary discussion**

- Introduce interviewers
- Thank you for being able to help this research. Before we get into the detail, can we just first deal with the administrative side of things?
- [Zoom recording, consent and confidentiality discussion]

Thank you very much for agreeing to take part in this interview about voluntary assisted dying. The purpose of the interview is to hear from you, in your own words, your experiences [or those of your family member] of making decisions about voluntary assisted dying. There is a chance you may feel that some of the questions asked are stressful or upsetting. If you do not wish to answer a question, you may skip it and go to the next question, or you may take a break. You may find some questions difficult to answer, however we are interested in whatever information you are able to provide. Please answer the questions in as much or as little detail as you like. Do you have any questions for me before we start the interview?

**About the person seeking VAD (introduction)**

If patient

Before we go on to talk about your experiences of making decisions about voluntary assisted dying, would it be OK if we asked about your illness and where you are now? Are you okay with that?

- Prompts: if appropriate, a chance to understand a bit about their approach to managing their illness and decision-making.

If family

Before we go on to talk about your experiences of making decisions about voluntary assisted dying, would it be possible if you could maybe just give us a short picture of your [family member]? We will talk about this some more – but just a nutshell, even a minute or two, just to help us get to know [family member] first.

**Remaining interview approach**

In terms of our discussion, I know we have a lot to cover and I want to make sure we can capture it all. I have found it often helps if I maybe step through the VAD process in order so you can comment on each part of the process. But I want to finish with some more general and open-ended questions which will also provide a chance for you to tell me other things. Is it OK if we proceed that way and perhaps if am a bit directive to make sure we understand your experience?

[Be prepared though if participant wants to talk about general things first – in which case start with the general questions from the end]

**First discussion of VAD (and first request)**

- When did you first become aware of VAD and that it might be possible? How did this topic come up? (e.g. sources of information) Who did you talk to about this?
- Was it easy to get information you wanted about VAD or were there barriers? Where did you go for information?
- When did you first talk about VAD with a doctor or health professional? How was discussion of this topic received? Were there any barriers or concerns with your doctor or other health professionals?
- Did you know that health professionals can’t raise VAD with you first? What do you think of that law?

**Assessment process [first assessment, second assessment, appointing a contact person, written declaration, permit]**

- What is your recollection of how your eligibility for VAD was assessed?
  - Prompts: Was it straightforward? Challenging? Why? Were any eligibility criteria difficult to satisfy?
  - Who helped and who hindered?
- What parts of the process worked well?
- What parts of the process needed improvement?
- General prompts
  - How did you find the VAD Care Navigator service?
  - Did you ever use telehealth for VAD consultations? Were there issues with travel to avoid telehealth?
  - Did your facility facilitate access to VAD or was it a barrier to access? How?
  - Were you able to find a doctor to coordinate the process, and then a second doctor?
  - Were you able to find witnesses to witness the written declaration?
  - Were you aware that your doctor had to obtain a permit? How did you find the permit process?

**Prescription of medication and provision of VAD for eligible VAD patients [prescription process, self-administration vs practitioner-administration, after death care]**

- Was there a discussion about which method of VAD to use? [self or practitioner administration] Would you have preferred a choice?
- What was your experience of obtaining the medication?
  - Prompts: Was it straightforward? Challenging? Why?
- What aspects of the process for getting the medication work well and what needs improvement?
- How long did the process take from when you started seeking VAD to when you had access to the medication? (explore time from when made formal first request to access to medication as well)
- General prompts
  - What was your experience with the Statewide Pharmacy service?
  - Did your facility facilitate access to VAD or was it a barrier to access? How?

Family members additionally asked these questions:

- What was your experience of accessing and using the medication? [looking for system issues, not clinical ones]
  - Prompts: Was it straightforward? Challenging? Why?
  - Prompts: Preparing the medication, experience of person taking it or doctor administering it
- How did the VAD system work after [family member’s death]?
  - Prompts: Who was the contact person? Why where you/they appointed? How did you/they find undertaking that role? Other issues?
- After the process was all finished, did you have any contact with anyone about how the VAD system worked or initiate yourself providing any feedback (positive or negative)? How it was handled by the institution/health professional?
- Once family member got the medication, how did they feel about that? Why did they go down this path of seeking VAD? (motivation, what they wanted)
- The choice about whether or not to take it, and if did, when to take it. How did they make these decisions and what guided them about the decision to take the medication and its timing?

Patients additionally asked these questions:

- Who is your contact person and why did you appoint them?
- If has medication/or if getting medication: Now that you have the medication (if have), how do you feel about that? Why did you go down this path of seeking VAD? (motivation, what they wanted)
- If has medication/or if getting medication: now/if that you have the medication, the choice about whether or not to take it, and if you do, when to take it – this choice is yours. How will you make these decisions and what will guide you about if and when?
- If you do take the medication, do you have any plans in terms of how that will occur? Who present, how happen, process.

**Sources, operation, navigation and integration of VAD regulation**

- How did you know what process to go through in seeking VAD?
- What did you do, or would you do, if you were unsure of the process or what was permitted or not permitted? Where would you look (or did look) or who would you (or did you) ask? What was your role/your family member’s role in progressing the process – who was “driving” the procedural aspects: the health professionals, patient, family?
  - Prompts: Guided by doctor, VAD Care Navigators, contact point at hospital, advocacy group, other?
  - Prompts: If talk about information sheets or policies etc, how useful?
- Were there any roadblocks? Disagreements? Difficulties accessing VAD? What did you do to get past this?
  - Prompt: Explore especially if one or more doctors refused eligibility for VAD or to participate as will need to navigate system more by themselves.
  - Prompt (if not covered above): Did your facility facilitate access to VAD or was it a barrier to access? How?
  - Prompt (if disagreement or barrier): Explore if they initiated any complaint processes formal or informal and how they did that.
- What would have helped to make navigating the VAD system easier? Prompt: Not just individuals but what is missing in the system?

**Perceptions about VAD regulation generally**

- What was the biggest challenge you faced in navigating the VAD system? How could current system of VAD be improved? What are barriers?
- What was the thing that worked best with the current system of VAD? What other parts of the system worked well? [explore key factors here]
- We have spoken a lot about the *processes* of VAD but what about the rules about who can have access to it in the first place (explain eligibility criteria if needed). Is this the right group who should have access to the law? (especially discuss with patients found not eligible)
- VAD involves a system that has safeguards to ensure only those who are eligible have access to VAD (safeguarding vulnerable and wider community) while facilitating access for those who qualify (choice for terminally ill patients). So a balance between safe processes that ensure only eligible people access VAD – but a system that is workable so people can in fact access VAD. How do you think the current VAD system strikes this balance?
  - From your experience, did you think the processes in the system fulfilled their role to keep ineligible people out? Or did they make it too hard to get through?
- Putting aside the Victorian system, if you could tell those designing a new VAD system who wanted it to be the best system possible, what advice would you have?
  - What principles or values do you think should underpin that system?
  - What features do you think the system should have?
  - This is about guiding people’s behaviour – what is the most effective way to guide people’s in this area?
- Is there anything else you wanted to mention?

**Demographics**

For this research to properly understand how the VAD system is working, we need to make sure we talk to people with diverse experiences and backgrounds. Would it be OK to ask you some questions about yourself and your care (or your family member) so we can understand your perspectives on what we discuss? Apologies if this is a bit mechanical and we will skip over what we already know from our discussion.

Patient

- Age (or age at death)
- Gender
- Where live (city or town)
- Illness type
- Other relevant medical conditions (i.e. comborbidities)
- Cultural aspects can be important to understand in this area [and relevant for eligibility criteria], so also asking participants about ethnic or cultural background, including country of birth. Also ask if any religious identity.
- Primary place where you/family member are/were cared for [make sure includes location VAD received if not in primary place]:
  - Hospital/hospice/RACF/at home/other
  - Public and private sector
  - Religious institution (if so, what religion)
- When sought VAD (rough timing: aiming to know if in first year of system or after that)
- Approved for VAD and used it/ Approved for VAD but didn’t use it/ Not approved for VAD (e.g. died during process or found to be ineligible)
- Relationship/marital status
- Family around patient and involvement in VAD (very brief but aiming to know if support (or opposition) around and who was involved in process)
- Highest level of education
- Occupation

Family member

- Date of birth/age
- Gender
- Relationship with patient [if not already mentioned]

**Wrapping up**

[Discussion about opportunity to review transcript; receiving findings; check-in about well-being and supports/resources for participants]

**Canada - caregivers**

**OVERVIEW OF INTERVIEW GUIDE**

**- Caregivers -**

**Introduction**

- Introduce interviewer(s) and study.
- Thank you for being able to help with this research. Before we go into detail, can we just first deal with the administrative side of things?
- [Zoom recording, consent, and confidentiality discussion].
- You may feel that some of the questions I ask are stressful or upsetting. If you do not want to answer any of these questions, please just say so. There are no right and wrong answers; we are simply interested in your views.
- Do you have any questions for me before we start the interview?

**Overview of individual seeking MAiD**

Before we go on to talk about your experiences of MAiD and how the process went for your family member, would it be possible to give us a short picture of your family member? We will talk about this some more – but just a nutshell, even a minute or two, just to help us get to know [family member] first.

**Remaining interview approach**

In terms of the rest of our discussion, I know we have a lot to cover and I want to make sure we can capture it all. To start let’s step through the MAiD process in order so you can comment on each part of the process. I will then finish with some more general and open-ended questions which will also provide a chance for you to tell me other things. Is it OK if we proceed that way and perhaps if am a bit directive to make sure we understand your experience?

**First discussion of MAiD**

- What was the extent of your exposure to MAiD (if any) prior to your experience with your family member?
- When did your family member first become aware of MAiD and that it might be possible? How did this topic come up (e.g. sources of information)?
  - Prompts: Who did they talk to about this? Were you involved in these conversations?
  - Why did you/they go down this path of seeking information about MAiD? (Motivation, what they wanted).
  - If individual or family member was involved in prior MAiD advocacy or reform, ask further information about nature of involvement and motivations for involvement in this work.
- Was it easy to get information about MAiD or were there barriers?
  - Prompt: Where did you/they go for information?
- When did they first talk about MAiD with a physician or nurse practitioner (or other health professional/care coordinator)? Who raised it? How did the health professional respond?
  - Prompt: Were there any barriers or concerns with their physician, nurse practitioner, or other health professionals?

**Assessment process**

- Can you tell us when your family member sought MAiD [and which track they were on (if post March 2021)]?
- What was their experience of requesting and being assessed for MAiD? How did the process start?
  - Prompts: Were they able to easily find a physician/nurse practitioner to guide them through the process? Were they connected to a care coordinator or other service?
  - Can you recall whether they had to make a written request to a physician or nurse practitioner to start the process? How did this work?
  - Were there any difficulties in getting a witness (or two if pre-March 2021) for this written request?
  - Who located the health professionals to do the assessments? Were there any difficulties in finding two independent physicians/nurse practitioners to confirm they were eligible?
  - Were there any difficulties with eligibility criteria? [Particularly ask about specific eligibility criteria if mentioned as an issue].
    - [Note after March 2021, reasonably foreseeable natural death is no longer an eligibility criterion but does determine whether a person is Track 1 or Track 2].
  - Do you recall how long the process took from the written request to when they were found eligible for MAiD?
- General prompts:
  - Did you/they need to contact any care coordination services?
  - Was telehealth ever used for MAiD consultations?
  - Were there issues with travel to access health professionals for assessments or for the provision of MAiD?
  - Where were they living when seeking MAiD? Did their facility facilitate access to MAiD or was it a barrier to access? How? Was this explicit or implicit? [e.g. institutional objection].
  - What did they think about the waiting period? [Note: prior to March 2021, 10-day waiting period and after March 2021, 90-day period for Track 2 (when natural death not reasonably foreseeable), but potential to be shortened if loss of capacity imminent].
  - [If Track 2 (post March 2021, natural death is not reasonably foreseeable) and neither assessor/providers have expertise in the illness/condition]: Were there any difficulties finding an independent practitioner with expertise in the condition causing the person’s suffering? [Note this would often be arranged by clinicians/care coordinators].
- Overall, looking back at the request and assessment process, was the process straightforward? Challenging? Why?
  - Prompts: What parts of the process worked well? What parts (if any) needed improvement?

**Prescription of medication and provision of MAiD for eligible MAiD patients**

If provider administered:

- Did your family member have provider-administered MAiD? Was there any discussion of self-administration as an alternative? If so, what were you/they told and what impression were you left with about it?
- Can you walk us through what happened from when the provider arrived until when your family member passed away?
  - Prompts: How long did the process take from when they started seeking MAiD to when your family member had chosen a date to receive MAiD?
  - The choice about whether or not to have MAiD and when to have it – that was a choice. What was guiding/guided them about the decision to have MAiD when they did?
  - Looking back, what aspects of the process for MAiD provision work well and what needs improvement?
  - [If after March 2021 and Track 1 (natural death reasonably foreseeable)] Did they consider a final consent waiver?
  - What was their experience of this (in terms of making the written arrangement and/or in the arrangement being followed)?
  - [If not at home] Did their facility facilitate the MAiD provision or did the facility create any barriers? How? Explicit or implicit? (E.g. institutional objection).
  - What was the process after your family member’s death?

If self-administered:

- How was the process of obtaining and using the medication? Can you walk us through what happened from when your family member obtained the medication until when they passed away?
  - Prompts: any involvement in preparing the medication, experience of person taking it, experience with pharmacists.
  - Once they got the medication, how did they feel about that?
  - The choice about whether or not to take it, and if they did, when to take it – that was a choice. What was guiding/guided them about the decision to take the medication and its timing?
  - Looking back, what aspects of the process for getting the medication/using MAiD medication work well and what needs improvement?
  - [If not at home] Did their facility facilitate access or was it a barrier? How? Explicit or implicit? (E.g. institutional objection).
  - Prompts: prescription process, self-administration, final consent (or advance consent)
  - What was the process after your family member’s death?

**Sources, operation, navigation and integration of MAiD regulation**

- [Looking back at whole process].
- How did you/your family member know what steps you needed to go through in seeking MAiD? For example, if you/they were not sure about what came next in the process, what did you do?
  - Prompts: guided by physician/nurse practitioner, care coordination service, others?
  - Prompts: What was your role/your family member’s role in progressing the process – who was ‘driving’ the procedural aspects of the process: the health professionals, patient, family? To what extent did you/your family member feel like you/they were in control of the process?
- What did you/they do, or would you/they do, if you/they were unsure of the process or what was permitted or not permitted? Where would you/they look (or did look) or who would you/they (or did you) ask?
  - Prompt: Guided by physician, care groups, contact point at hospital, advocacy group, other?
  - Prompts: If participant discusses information sheets or policies etc. ask whether useful and why.
- Were there any roadblocks? Disagreements? Difficulties accessing MAiD? What was done to get past this?
  - Prompt: Explore especially if one or more physician/nurse practitioners/pharmacists refused to participate.
  - Prompt (if not covered): [If in an institutional setting] - Did your facility facilitate access to MAiD or was it a barrier to access? How? Explicit or implicit? (e.g. Institutional objection).
  - Prompt (if disagreement or barrier): Explore if they initiated complaint process, formal or informal, and how this was initiated.
- What would have helped to make navigating the MAiD system easier?
  - Prompt: Not just individuals, but what is missing in the MAiD system?
- After the process was finished, did you have any contact with anyone about how the MAiD system worked? Did you provide any feedback (positive or negative)? E.g. how it was handled by institution or health professional?
- Have you had any follow up in terms of support with grief and bereavement?
- How has your experience with MAiD and any grief/bereavement shaped or changed your behaviour and attitudes?
  - Prompt: Some participants feel motivated to be involved in advocacy after their experience, whereas others feel quite isolated and as though they can’t speak about it. Where do you sit?

**Perceptions about MAiD regulation generally**

- [Shifting to some more big picture questions now].
- Overall, what was the biggest challenge you/your family member faced in navigating the MAiD system?
  - Prompt: If you could fix one thing with the current system, what would it be?
- Overall, what was the best thing in the MAiD system process?
  - Prompts: What was the key part of the process? If there was one thing that you think is critical for the system running well, what is that?
- We have spoken a lot about the *processes* of MAiD but what about the rules about who can have access to it in the first place (explain eligibility criteria if required). Is this the right group who should have access? (Especially discuss with individuals/families of patients found ineligible).
- MAiD involves a system that has safeguards to ensure only those who are eligible have access to MAiD (safeguarding vulnerable and wider community) while facilitating access for those who qualify (a choice for persons who are suffering from a grievous and irremediable medical condition). So, there is a balance between safe processes that ensure only eligible people access MAiD – but a system that is workable so people can in fact access MAiD. How do you think the current MAiD system strikes this balance?
  - From your experience, is access too easy or too hard, or appropriate?

**Demographic information about individual who sought MAiD**

For this research to properly understand how the MAiD system is working, we need to make sure we talk to people with diverse experiences and backgrounds. Would it be OK to ask you some questions about yourself and your family member so we can understand your perspectives on what we discuss? Please feel free to say “pass” on any you don’t wish to answer.

Questions about participant

- Age
- Gender
- Relationship to family member/patient [if not already mentioned]

Questions about family member/patient

- Age
- Gender
- Marital/relationship status
- Highest educational level
- Occupation
- Country of birth
- Location of residence (e.g. city, town, rural)
- Illness, disease or medical condition
- Other relevant medical conditions
- Cultural background (including ethnicity, religion, if comfortable sharing)
- Primary place of care (and place of administration, if different)
- When MAiD sought
- Outcome of MAiD

**Wrapping up**

[Discussion about opportunity to review transcript; receiving findings; check-in about well-being and supports/resources for participants]

**Canada - patients**

**OVERVIEW OF INTERVIEW GUIDE**

**- Patients -**

**Introduction**

- Introduce interviewer(s) and study.
- Thank you for being able to help with this research. Before we go into detail, can we just first deal with the administrative side of things?
- [Zoom recording, consent, and confidentiality discussion].
- You may feel that some of the questions I ask are stressful or upsetting. If you do not want to answer any of these questions, please just say so. There are no right and wrong answers; we are simply interested in your views.
- Do you have any questions for me before we start the interview?

**Overview of individual seeking MAiD**

Before we go on to talk about your experiences of making decisions about MAiD and the process, can we just briefly talk about your illness and where you are now? Are you okay with that? [If appropriate, a chance to understand a bit about the person’s approach to managing their condition and decision-making].

**Remaining interview approach**

In terms of the rest of our discussion, I know we have a lot to cover, and I want to make sure we can capture it all. To start let’s step through the MAiD process in order so you can comment on each part of the process. I will then finish with some more general and open-ended questions which will also provide a chance for you to tell me other things. Is it OK if we proceed that way and perhaps if am a bit directive to make sure we understand your experience?

**First discussion of MAiD**

- What was your exposure to MAiD (if any) prior to your own experience?
- When did you first become aware of MAiD and that it might be possible? How did this topic come up (e.g. sources of information)?
  - Prompts: Who did you talk to about this?
  - Why did you go down this path of seeking information about MAiD? (Motivation, what you wanted).
  - [If involved in prior MAiD advocacy or reform, ask further information about nature of involvement and motivations for involvement in this work].
- Was it easy to get information you wanted about MAiD or were there barriers?
  - Prompt: Where did you go for information?
- When did you first talk about MAiD with a physician or nurse practitioner (or other health professional/care coordinator)? Who raised it? How did the health professional respond?
  - Prompt: Were there any barriers or concerns with your physician, nurse practitioner, or other health professionals?

**Assessment process**

- Can you tell us when you sought MAiD [and which track you were on (if post March 2021)]?
- What was your experience of requesting and being assessed for MAiD? How did the process start?
  - Prompts: Were you able to easily find a physician/nurse practitioner to guide you through the process? Were you connected to a care coordinator or other service?
  - Can you recall whether you had to make a written request to a physician or nurse practitioner to start the process? How did this work?
  - Were there any difficulties in getting a witness (or two if pre-March 2021) for this written request?
  - Who located the health professionals to do the assessments? Were there any difficulties in finding two independent physicians/nurse practitioners to confirm you were eligible?
  - Were there any difficulties with eligibility criteria? [Particularly ask about specific eligibility criteria if mentioned as an issue].
    - [Note after March 2021, reasonably foreseeable natural death is no longer an eligibility criterion but does determine whether a person is Track 1 or Track 2].
  - Do you recall how long the process took from the written request to when you were found eligible for MAiD?
- General prompts:
  - Did you need to contact any care coordination services?
  - Did you ever use telehealth for MAiD consultations?
  - Were there issues with travel to access health professionals for assessments or for the provision of MAiD?
  - Where were you living when seeking MAiD? Did your facility facilitate access to MAiD or was it a barrier to access? How? Was this explicit or implicit? [e.g. institutional objection].
  - What do you think about the waiting period? [Note prior to March 2021, 10-day waiting period and after March 2021, 90-day period for Track 2 (when natural death not reasonably foreseeable), but potential to be shortened if loss of capacity imminent].
  - [If Track 2 (post March 2021, natural death is not reasonably foreseeable) and neither assessor/providers have expertise in the illness/condition]: Were there any difficulties finding an independent practitioner with expertise in the condition causing your suffering to confirm eligibility? [Note this would often be arranged by clinicians/care coordinators].
- Overall, looking back at the request and assessment process, was the process straightforward? Challenging? Why?
  - Prompts: What parts of the process worked well? What parts (if any) needed improvement?

**Prescription of medication and provision of MAiD for eligible MAiD patients**

If plan to have provider administered MAiD:

- Will you choose to have provider-administered MAiD? Was there any discussion of self-administration as an alternative? If so, what were you told and what impression were you left with about it?
- The choice about whether or not to have MAiD it and when to have MAiD – that is a choice. What is guiding you about your decision to have MAiD and the timing of that?
- [If after March 2021 and Track 1 (natural death reasonably foreseeable)]: Have you considered a final consent waiver? What was your experience of this (in terms of making the written arrangement)?

If plan to have self-administered MAiD:

- When will you obtain the medication?
  - How do you think you will feel when you obtain it?
- Have you considered advance consent for failed self-administration? What was your experience of this (in terms of making a written arrangement)?

**Sources, operation, navigation and integration of MAiD regulation**

- [Looking back at whole process].
- How did you know what steps you needed to go through in seeking MAiD? For example, if you were not sure about what came next in the process, what did you do?
  - Prompts: guided by physician/nurse practitioner, care coordination service, others?
- What did you do, or would you do, if you were/are unsure of the process or what was permitted or not permitted? Where would you look (or did look) or who would you (or did you) ask?
  - Prompt: Guided by physician, nurse practitioner, care groups, contact point at hospital, advocacy group, other?
  - Prompts: If participant discusses information sheets or policies etc. ask whether useful and why?
- Were there any roadblocks? Disagreements? Difficulties accessing MAiD? What was done to get past this?
  - Prompt: Explore especially if one or more physician/nurse practitioners/pharmacists refused to participate.
  - Prompt (if not covered): [If in an institutional setting] - Did your facility facilitate access to MAiD or was it a barrier to access? How? Was this explicit or implicit? (e.g. Institutional objection).
  - Prompt (if disagreement or barrier): Explore if they initiated complaint process, formal or informal, and how this was initiated.
- What would have helped to make navigating the MAiD system easier for you?
  - Prompt: Not just individuals, but what is missing in the MAiD system?
- Have you had any contact with anyone about how the MAiD system has worked so far? Did you provide any feedback (positive or negative)? E.g. how it was handled by institution or health professional?

**Perceptions about MAiD regulation generally**

- [Shifting to some more big picture questions now].
- Overall, what was the biggest challenge you faced in navigating the MAiD process?
  - Prompt: If you could fix one thing with the current system, what would it be?
- Overall, what was the best thing in the MAiD process?
  - Prompts: What was the key part of the process? If there was one thing that you think is critical for the system running well, what is that?
- We have spoken a lot about the *processes* of MAiD but what about the rules about who can have access to it in the first place (explain eligibility criteria). Is this the right group who should have access? (Especially discuss with individuals found ineligible).
- MAiD involves a system that has safeguards to ensure only those who are eligible have access to MAiD (safeguarding vulnerable and wider community) while facilitating access for those who qualify (a choice for people who are suffering from a grievous and irremediable medical condition). So, there is a balance between safe processes that ensure only eligible people access MAiD – but a system that is workable so people can in fact access MAiD? How do you think the current MAiD system strikes this balance?
  - From your experience, is access too easy or too hard, or appropriate?

**Demographic information about individual who sought MAiD**

For this research to properly understand how the MAiD system is working, we need to make sure we talk to people with diverse experiences and backgrounds. Would it be OK to ask you some questions about yourself so we can understand your perspectives on what we discuss? Please feel free to say “pass” on any you don’t wish to answer.

- Age
- Gender
- Marital/relationship status
- Highest educational level
- Occupation
- Country of birth
- Location of residence (e.g. city, town, rural)
- Illness, disease or medical condition
- Other relevant medical conditions
- Cultural background (including ethnicity, religion, if comfortable sharing)
- Primary place of care (and place of administration, if different)
- When MAiD sought
- Outcome of MAiD

**Wrapping up**

[Discussion about opportunity to review transcript; receiving findings; check-in about well-being and supports/resources for participants]
