# Supplementary material for: Patients' and Caregivers' Suggestions for Improving Assisted Dying Regulation: A Qualitative Study in Australia and Canada
Source: Health Expect. 2024 Jun 19;27(3):e14107. doi: 10.1111/hex.14107 (PMC11187863; doi:10.1111/hex.14107)
Supplement: Supplementary file 2 — Supporting information. [file HEX-27-e14107-s001.docx]

| Topic | Item No. | Guide Questions/Description | Reported  on Page No. | Additional comments |
| --- | --- | --- | --- | --- |
| **Domain 1: Research team and reflexivity** | | | | |
| *Personal characteristics* | | | | |
| Interviewer/facilitator | 1 | Which author/s conducted the interview or focus  group? | 5 |  |
| Credentials | 2 | What were the researcher’s credentials? E.g. PhD,  MD | N/A | These are noted in the title page of the  manuscript. |
| Occupation | 3 | What was their occupation at the time of the study? | N/A | These are noted in the title page of the  manuscript. |
| Gender | 4 | Was the researcher male or female? | N/A |  |
| Experience and training | 5 | What experience or training did the researcher have? | 5 | BPW, EC and JD sat in on early interviews conducted by RJ as part of  RJ’s research training. |
| *Relationship with participants* | | | | |
| Relationship established | 6 | Was a relationship established prior to study commencement? | N/A | Some participants were known to the research prior to study commencement and were contacted for recruitment due to having experiences or perspectives the research team was seeking for the research. In other instances, no relationship was established prior to study commencement, except for contact to organise interview logistics and confirm  participant consent. |
| Participant knowledge of the interviewer | 7 | What did the participants know about the researcher? e.g. personal goals, reasons for doing the research | N/A | Participants were informed about the names of the researchers in the team, their roles in the team, and the aims of the study via the consent form and participant information materials. Additional  information was provided upon request to |

|  |  |  |  | participants, either prior to the interview (e.g. via email) or during the interview (e.g. at the start of the interview or in response to questions asked by participants). Some participants sought further information about the research team, such as personal goals or motivations for undertaking the research, which was provided upon request but not proactively disclosed to  participants. |
| --- | --- | --- | --- | --- |
| Interviewer characteristics | 8 | What characteristics were reported about the interviewer/facilitator? e.g. Bias, assumptions, reasons and interests in the research topic | N/A | Information about the interviewers including interest in the research topic or bias was discussed if asked by participants, or used as an interview technique to probe areas of interest raised  by participants during interviews. |
| **Domain 2: Study design** | | | | |
| *Theoretical framework* | | | | |
| Methodological orientation and Theory | 9 | What methodological orientation was stated to underpin the study? e.g. grounded theory, discourse analysis, ethnography, phenomenology, content  analysis | 4-6 |  |
| *Participant selection* | | | | |
| Sampling | 10 | How were participants selected? e.g. purposive,  convenience, consecutive, snowball | 4-5 |  |
| Method of approach | 11 | How were participants approached? e.g. face-to- face, telephone, mail, email | N/A | Contact between participants and the research team generally occurred via email, with mail and telephone also being  used in a small number of cases. |
| Sample size | 12 | How many participants were in the study? | 6 | See also Table 1 and Table 2. |
| Non-participation | 13 | How many people refused to participate or dropped  out? Reasons? | N/A | No participants dropped out of the study  after participating in an interview. One |

|  |  |  |  | prospective participant in Australia died  before the scheduled interview. |
| --- | --- | --- | --- | --- |
| *Setting* | | | | |
| Setting of data collection | 14 | Where was the data collected? e.g. home, clinic,  workplace | 5 |  |
| Presence of non-  participants | 15 | Was anyone else present besides the participants  and researchers? | N/A | No one was present besides the  participants and researchers. |
| Description of sample | 16 | What are the important characteristics of the sample? e.g. demographic data, date | Table 1 and  Table 2 |  |
| *Data collection* | | | | |
| Interview guide | 17 | Were questions, prompts, guides provided by the  authors? Was it pilot tested? | 5 | See also Supplemental File 1. |
| Repeat interviews | 18 | Were repeat interviews carried out? If yes, how many? | N/A | No repeat interviews were carried out, however, one Australian participant’s  interview occurred over 2 dates. |
| Audio/visual recording | 19 | Did the research use audio or visual recording to  collect the data? | 5 |  |
| Field notes | 20 | Were field notes made during and/or after the  interview or focus group? | 5-6 |  |
| Duration | 21 | What was the duration of the interviews or focus  group? | 6 |  |
| Data saturation | 22 | Was data saturation discussed? | 4-5 | We use the term ‘information power’, reflecting our reflexive thematic analysis  methodology. |
| Transcripts returned | 23 | Were transcripts returned to participants for  comment and/or correction? | 5 |  |
| **Domain 3: Analysis and findings** | | | | |
| *Data analysis* | | | | |
| Number of data coders | 24 | How many data coders coded the data? | 5-6 |  |

| Description of the coding  tree | 25 | Did authors provide a description of the coding tree? | N/A | A description of the coding tree is not  provided. |
| --- | --- | --- | --- | --- |
| Derivation of themes | 26 | Were themes identified in advance or derived from  the data? | 5-6 |  |
| Software | 27 | What software, if applicable, was used to manage  the data? | 6 |  |
| Participant checking | 28 | Did participants provide feedback on the findings? | N/A | Participants were not provided with the opportunity to give feedback on the findings prior to submission of this  research article. |
| *Reporting* | | | | |
| Quotations presented | 29 | Were participant quotations presented to illustrate the themes/findings? Was each quotation identified?  e.g. participant number | Part 4 |  |
| Data and findings  consistent | 30 | Was there consistency between the data presented  and the findings? | Part 4 |  |
| Clarity of major themes | 31 | Were major themes clearly presented in the findings? | Part 4 |  |
| Clarity of minor themes | 32 | Is there a description of diverse cases or discussion  of minor themes? | Part 4 |  |

Developed from: Tong, A., Sainsbury, P., & Craig, J. (2007). Consolidated criteria for reporting qualitative research (COREQ): a 32-item checklist for interviews and focus groups. International Journal for Quality in Health Care, 19(6), 349-357. doi: 10.1093/intqhc/mzm042
